# CT-Based Evaluation of Sphenoid Sinus Type and Sella Turcica-Related Quantitative Parameters for Endoscopic Endonasal Transsphenoidal Approach Planning in Pediatric Patients

**DOI:** 10.3390/diagnostics16182958

**Published:** 2026-09-13

**Authors:** İbrahim Akbudak, Muhammed Tekinhatun

**Affiliations:** Department of Radiology, Faculty of Medicine, Dicle University Hospital, 21280 Diyarbakır, Turkey; mtekinhatun@gmail.com

**Keywords:** Pediatrics, computed tomography, sella turcica, endoscopic endonasal transsphenoidal approach

## Abstract

**Background:** To evaluate sphenoid sinus (SS) types and sella turcica (ST)-related quantitative parameters on contrast-enhanced computed tomography (CECT) images according to age and sex in pediatric patients, and to assess their relevance to endoscopic endonasal transsphenoidal approach (EETA) planning. **Methods:** This retrospective study included 254 patients younger than 18 years without sellar or SS pathology. Patients were categorized according to age and sex. The following parameters were assessed on CECT images: SS type, skull base angle, anterior surgical angle (ASA), posterior surgical angle (PSA), anterior distances (AD1–2), posterior distances (PD1–2), sella turcica depth (DST), intercarotid distance (ID), safe window area (SWA), apex nasi–sella turcica angle (ANSTA), thickness of the sella turcica anterior wall (TSAW), and thickness of the sella turcica floor wall (TSFW). **Results:** The mean age was 9.72 ± 5.03 years. Although the conchal SS type was most frequent among patients aged 0–6 years, the sellar and postsellar types increased with advancing age. With advancing age, ASA, PSA, AD1–2, PD1–2, DST, ID, and SWA increased, whereas TSAW decreased. In unadjusted comparisons, all evaluated quantitative parameters differed significantly among SS types (*p* < 0.05). SWA showed significant positive correlations with ASA, PSA, AD1–2, PD1–2, and ANSTA, and a significant negative correlation with TSAW (*p* < 0.001). **Conclusions:** Pediatric SS and ST anatomy show substantial age-related changes. Assessment of CECT-based quantitative parameters may help define the sellar corridor, estimate the available working area, and support EETA planning.

## 1. Introduction

The sella turcica (ST) is a saddle-shaped anatomical structure located in the central portion of the sphenoid bone that houses the pituitary gland. The morphology and anatomical characteristics of the ST vary across different age groups and ethnic populations [1].

The endoscopic endonasal transsphenoidal approach (EETA) is a well-established technique for the treatment of sellar and parasellar lesions in adults [2]. However, its use in pediatric patients remains technically challenging because of age-related anatomical limitations, including narrow intercarotid distance, absent or limited sphenoid sinus (SS) pneumatization, and small nasal cavity dimensions. Although craniotomy was traditionally preferred for pediatric sellar and skull base lesions, EETA offers important advantages, such as reduced brain retraction-related tissue injury and preservation of facial and dental development [3]. During EETA, the sellar wall is reached through the nasal cavity and SS; however, the limited surgical corridor and complex parasellar anatomy increase the risk of serious complications, particularly internal carotid artery injury and cavernous sinus hemorrhage [4].

The morphology and dimensions of the SS vary according to ethnicity, age, and sex [5]. Although SS is generally non-pneumatized at birth, pneumatization begins in early childhood. It has been reported that the rate of SS pneumatization accelerates between the ages of 3–5 and 7–12 [6]. SS pneumatization generally reaches adult size at around 12–14 years of age [7]. However, another study has indicated that pneumatization reaches full development between the ages of 15 and 30 [8]. According to the extent of pneumatization, the SS is classified as conchal, presellar, sellar, or postsellar: the conchal type does not reach the anterior wall of the ST, the presellar type extends to this wall, the sellar type extends between the anterior and posterior ST walls, and the postsellar type extends beyond the posterior ST wall [9,10]. Because computed tomography (CT) enables detailed evaluation of SS anatomy, preoperative CT assessment is essential before EETA [11,12].

Before EETA, detailed radiological evaluation of the nasal cavity, SS, ST, parasellar region, and adjacent vital vascular and neural structures is essential for identifying relevant anatomical features and variations. Although several anatomical parameters relevant to EETA have been investigated in adult populations, comprehensive pediatric data evaluating SS type together with ST-related distance, angle, area, and wall-thickness parameters on CECT remain limited. Therefore, the aim of this study was to determine SS types and evaluate ST-related area, distance, and angle parameters on contrast-enhanced computed tomography (CECT) images in pediatric patients, with the goal of contributing to EETA planning and potentially aiding in the reduction in intraoperative complications.

## 2. Materials and Methods

This retrospective study was approved by the Institutional Ethics Committee of our institution (approval date/reference number: 12 June, 2024/200), and the requirement for informed consent was waived because of the retrospective study design. A total of 278 pediatric patients who underwent contrast-enhanced computed tomography (CECT) of the head and/or neck between 1 January 2021 and 1 July 2024, with imaging coverage including the nasal cavity, sphenoid sinus (SS), sella turcica (ST), and parasellar region, were initially reviewed. All patients were younger than 18 years of age. Cases were selected using a consecutive sampling method from patients with available CECT images in the picture archiving and communication system (PACS). All CECT examinations were performed as part of routine care based on clinical indications, including suspicion or evaluation of head–neck region mass lesions, infection/abscess, vascular malformations, trauma-related vascular complications, and complicated orbital diseases. No additional imaging or radiation exposure was administered for research purposes. In this retrospective analysis, CECT also allowed detailed assessment of both osseous sinonasal/sellar anatomy and adjacent vascular structures relevant to EETA planning.

Patients with space-occupying lesions involving the ST or SS and/or a history of surgery in these regions (n = 6), those with a history of surgery involving adjacent cranial structures or cranial disorders and malformations that could alter the regional anatomy (n = 7), and those with motion- or technique-related imaging artifacts (n = 11) were excluded from the study. Consequently, 254 patients were included and stratified according to sex and age. Based on previous studies, patients were categorized into five age groups: 0–2, 3–6, 7–11, 12–14, and 15–17 years [6,7,8].

All CECT examinations were performed using a dual-source CT scanner with 128 × 2 detector rows and two X-ray tubes positioned at an angle of 95° (Somatom Definition Flash; Siemens Healthcare, Erlangen, Germany). Imaging was performed using a pediatric protocol adjusted according to patient age and body habitus (temporal resolution, 75 ms; tube voltage, 70–120 kV; slice thickness, 0.6 mm). Images were reconstructed with a 512 × 512 matrix, I31s or J30f kernels, and a patient size-adjusted field of view (180–390 mm). Measurements were performed on bone window images (level, 400–500 HU; width, 2000–3000 HU). A nonionic iodinated contrast agent (iohexol, 300–350 mg/mL) was administered at a dose of 1–2 mL/kg using an automated injector system (CT Motion; Ulrich Medical, Ulm, Germany) at an injection rate of 0.5–5 mL/s. Following CECT acquisition, patients were monitored for at least 30 min for potential adverse reactions related to contrast administration.

### 2.1. Assessment of Sella Turcica-Related Parameters and Sphenoid Sinus Types

**Figure 1:**
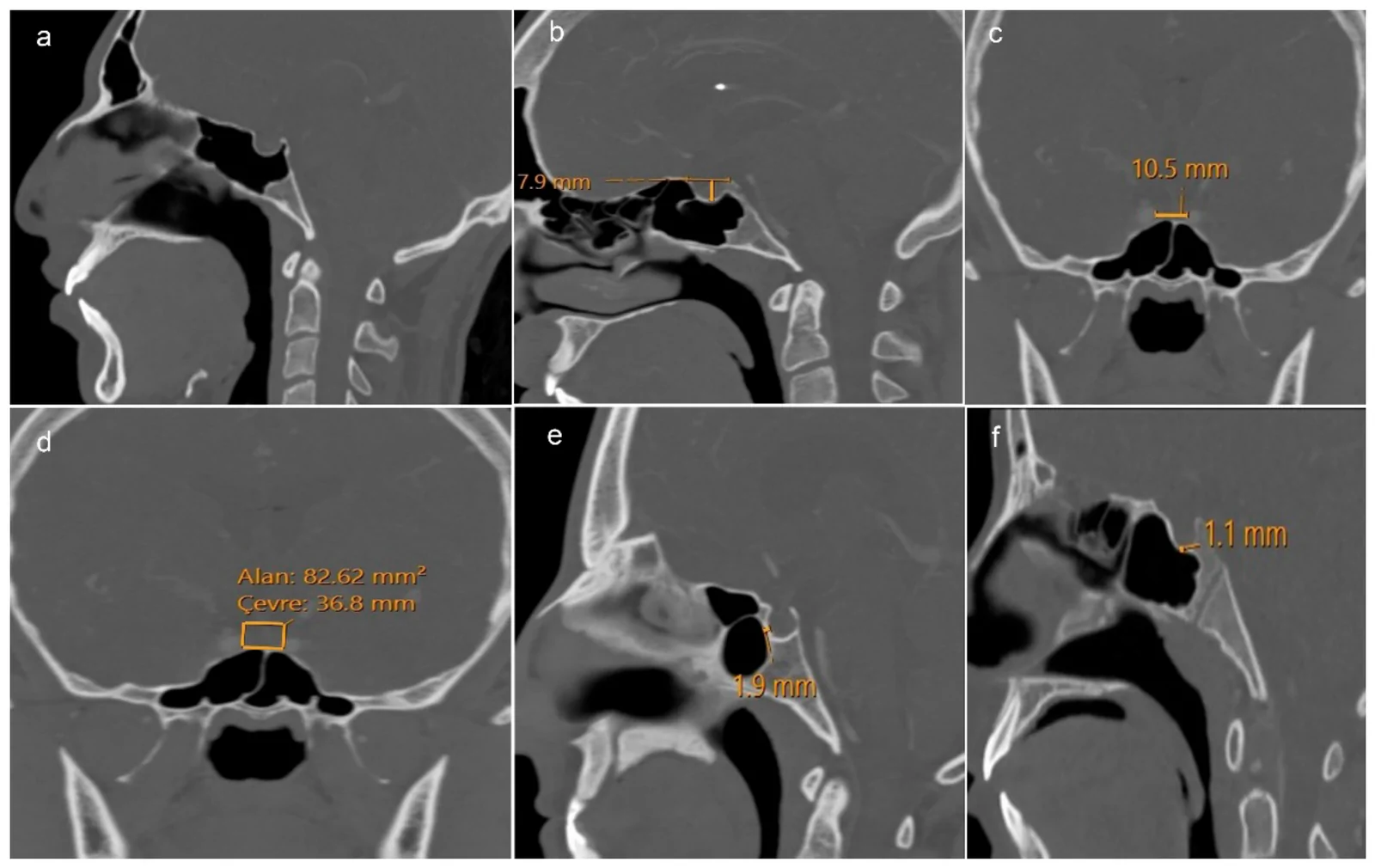
Coronal and sagittal CECT images of the sellar region. (**a**) Alignment of sellar anatomical structures with the anterior nasal spine on the sagittal image; (**b**) depth of the sella turcica (DST), 7.9 mm; (**c**) intercarotid distance (ID), 10.5 mm; (**d**) safe window area (SWA), 82.62 mm^2^; (**e**) thickness of the sella turcica anterior wall (TSAW), 1.9 mm; and (**f**) thickness of the sella turcica floor wall (TSFW), 1.1 mm.

**Figure 2:**
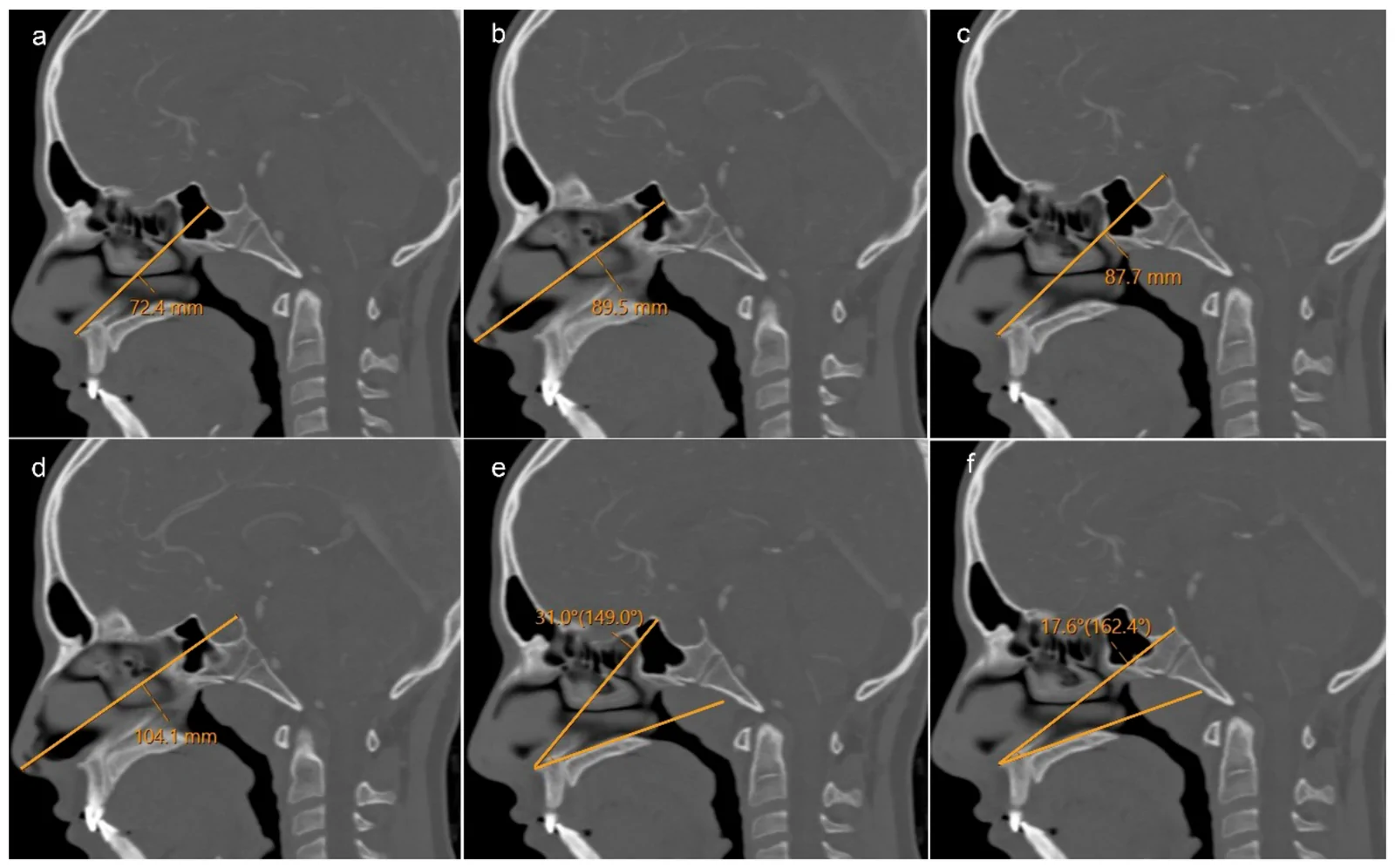
Sagittal CECT images of the sellar region. (**a**) Anterior distance 1 (AD1), 72.4 mm; (**b**) anterior distance 2 (AD2), 89.5 mm; (**c**) posterior distance 1 (PD1), 87.7 mm; (**d**) posterior distance 2 (PD2), 104.1 mm; (**e**) anterior surgical angle (ASA), 31.0°; and (**f**) posterior surgical angle (PSA), 17.6°.

**Figure 3:**
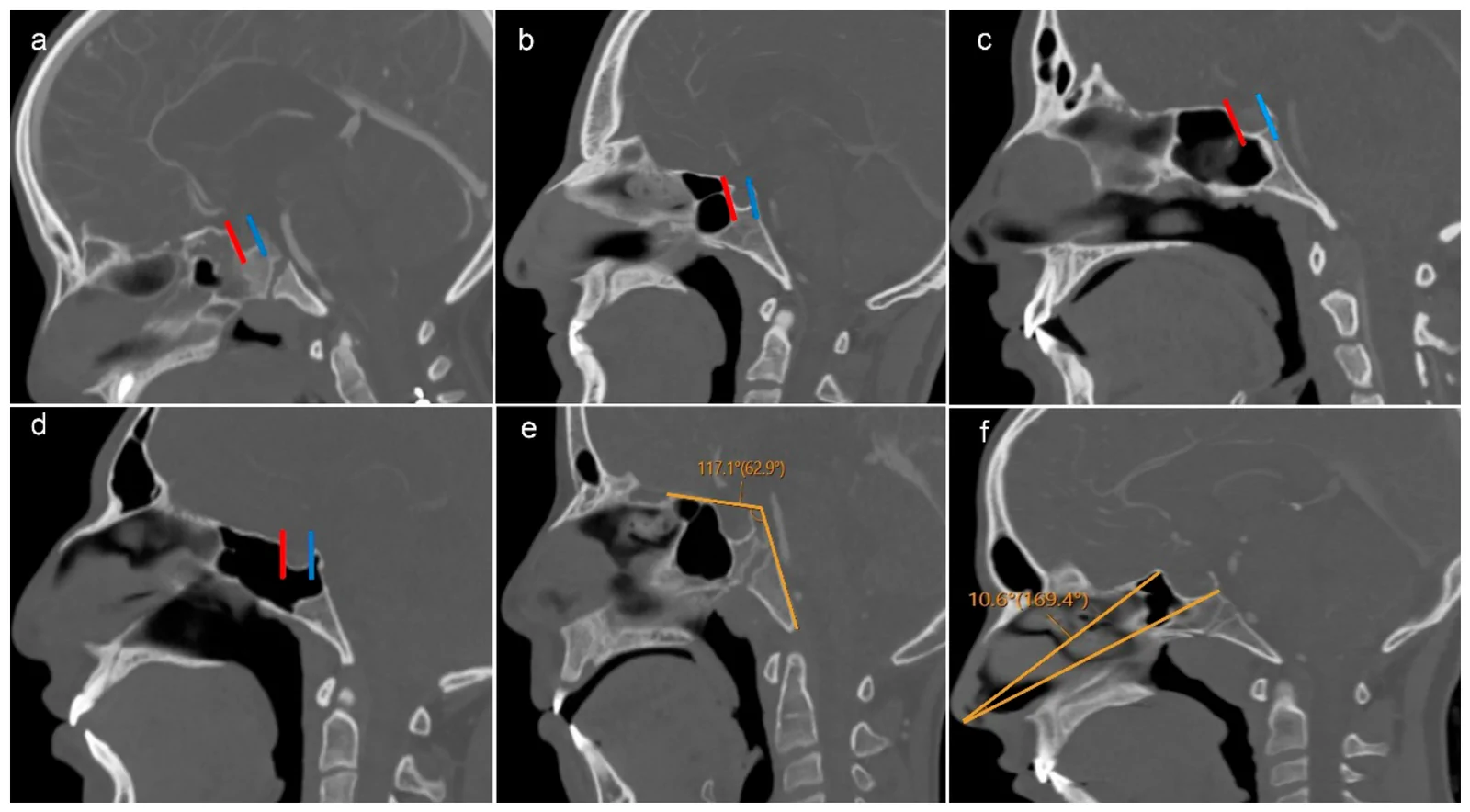
Sagittal CECT images of the sellar region. (**a**–**d**) Sphenoid sinus types; red line, anterior wall of the sella turcica; blue line, posterior wall of the sella turcica; (**a**) conchal type; (**b**) presellar type; (**c**) sellar type; (**d**) postsellar type; (**e**) skull base angle (SBA), 117.1°; and (**f**) apex nasi–sella turcica angle (ANSTA), 10.6°.

The skull base angle (SBA) was defined as the angle formed between lines drawn along the planum sphenoidale and the clivus. The anterior surgical angle (ASA) was defined as the angle between a line drawn from the anterior nasal spine to the superior margin of the anterior wall of the ST and a line parallel to the hard palate. The posterior surgical angle (PSA) was defined as the angle between a line parallel to the hard palate and a line extending from the anterior nasal spine to the inferior margin of the posterior wall of the ST. The apex nasi–sella turcica angle (ANSTA) was defined as the angle formed by lines drawn from the inferior aspect of the apex nasi to the superior margin of the anterior wall and the inferior margin of the posterior wall of the ST. Anterior distance 1 (AD1) was defined as the linear distance between the anterior nasal spine and the midpoint of the anterior wall of the ST. Anterior distance 2 (AD2) was defined as the linear distance between the inferior aspect of the apex nasi and the midpoint of the anterior wall of the ST. Posterior distance 1 (PD1) was defined as the linear distance from the anterior nasal spine to the superior margin of the posterior wall of the ST, whereas posterior distance 2 (PD2) was measured as the linear distance between the superior margin of the posterior wall of the ST and the inferior aspect of the apex nasi. The depth of the sella turcica (DST) was defined as the maximum perpendicular distance from the sellar floor to a line connecting the superior margins of the anterior and posterior walls of the ST. The intercarotid distance (ID) was measured as the shortest linear distance between the medial walls of the bilateral internal carotid arteries (ICAs) at the sellar level. Safe window area (SWA) was calculated using the ID x DST formula and expressed in square millimeters. The thickness of the sella turcica anterior wall (TSAW) and the thickness of the sella turcica floor wall (TSFW) were measured as the osseous thicknesses of the anterior wall and floor of the ST, respectively [4,13,14] (Figure 1, Figure 2 and Figure 3).

The SS was classified into four types according to the extent of pneumatization, as previously described in the literature: conchal, presellar, sellar, and postsellar [9,10] (Figure 3). Based on a previously reported classification of the skull base angle (SBA), patients were categorized into three groups: type A (≤104°), type B (105–120°), and type C (≥121°) [15].

These SS types and anatomical parameters were systematically identified, measured, and evaluated on preoperative CECT images. The coronal plane was used for the assessment of ID, whereas the sagittal plane was used for all other distance and angular measurements. The sagittal plane was aligned using the anterior nasal spine as a reference to ensure that all relevant anatomical structures were visualized within the same section. All measurements were performed twice by an experienced head and neck radiologist, and the mean values were recorded in the database in millimeters.

To assess measurement reliability, intraobserver agreement (after an 8-week interval) was evaluated using repeated measurements by the same radiologist, and interobserver agreement was evaluated using independent measurements by a second radiologist in a randomly selected subset of 51 patients (20.1% of the cohort), including all SS types. The second observer’s measurements were used only for reliability assessment.

### 2.2. Statistical Analysis

Statistical analyses were performed using SPSS software (Statistical Package for the Social Sciences, version 22.0; SPSS Inc., Chicago, IL, USA). Categorical variables were expressed as numbers (n) and percentages (%), whereas continuous variables were presented as mean ± standard deviation (SD), median, and interquartile range (25th–75th percentile). The Pearson chi-square test was used to compare categorical variables between groups. The distribution of continuous variables was assessed using the Kolmogorov–Smirnov test. Comparisons between two groups were performed using the Mann–Whitney U test, whereas comparisons among more than two groups were conducted using the Kruskal–Wallis test. Comparisons of quantitative parameters among SS types were considered unadjusted analyses because no adjustment for age was performed. Associations between continuous variables were evaluated using Spearman correlation analysis. Measurement reliability was analyzed using single-measure absolute-agreement intraclass correlation coefficients (ICCs) for continuous variables and Cohen’s kappa statistics for categorical SS type classification. ICC and kappa values were interpreted according to standard thresholds. A two-sided *p* value of < 0.05 was considered statistically significant for all analyses. Because this was a retrospective study based on consecutive eligible cases, no a priori sample size calculation was performed. A post hoc sensitivity power analysis based on the final sample size of 254 patients showed that the study had 80% power to detect a correlation coefficient of approximately r = 0.175 and an effect size of approximately Cohen’s f = 0.219 for comparisons among the five age groups at an alpha level of 0.05.

## 3. Results

A total of 254 patients were included in the study, comprising 145 males and 109 females (42.9%). The mean age was 9.72 ± 5.03 years (range, 0.5–17 years). Of the study population, 33 patients (13.0%) were in the 0–2-year age group, 41 (16.1%) in the 3–6-year age group, 79 (31.1%) in the 7–11-year age group, 43 (16.9%) in the 12–14-year age group, and 58 (22.8%) in the 15–17-year age group. Analysis of SS types demonstrated that the sellar type was the most common (35.0%, n = 89), followed by the conchal (32.7%, n = 83), presellar (25.2%, n = 64), and postsellar (7.1%, n = 18) types. Regarding skull base angle (SBA) classification, 13.0% of patients were categorized as type A, 64.2% as type B, and 22.8% as type C.

Among the 254 included patients, the median (IQR) age was 10 (6–14) years. The median (IQR) values were 114.6° (109.1–119.2) for SBA, 30.6° (28.7–32.9) for ASA, 22.1° (20.1–24.2) for PSA, 67.7 mm (59.3–74.5) for AD1, 85.3 mm (73.6–93.1) for AD2, 79.0 mm (71.9–85.0) for PD1, 95.2 mm (87.2–103.5) for PD2, 86.7 mm^2^ (68.3–106.3) for SWA, 6.5 mm (5.6–7.1) for DST, 13.2 mm (11.4–15.5) for ID, 6.3° (5.7–7.0) for ANSTA, and 1.1 mm (0.9–5.8) for TSAW. TSFW could only be measured in the sellar and postsellar types and was available in 107 patients, with a median value of 0.9 mm (0.8–1.0). However, in patients with conchal or presellar SS types, it could not be measured because the sphenoid bone at the base of the sella is not pneumatized at all and remains entirely bony (Table 1).

Intraobserver reliability was good to excellent for the quantitative measurements, with ICC values ranging from 0.869 to 0.990. Interobserver reliability was also good to excellent, with ICC values ranging from 0.806 to 0.989. For categorical SS type classification, intraobserver and interobserver Cohen’s kappa values were 0.891 and 0.838, respectively, indicating almost perfect agreement.

Sex-based comparisons revealed no statistically significant differences in age-group distribution or SS type distribution between male and female patients (*p* = 0.760 and *p* = 0.138, respectively). However, analysis of continuous variables demonstrated significant sex-related differences in SBA, PD1, PD2, DST, ID, and ANSTA (all *p* < 0.05; Table 2).

Unadjusted comparisons according to SS type revealed significant differences in age-group distribution (*p* < 0.001). The conchal type was more frequently observed in younger age groups, whereas the sellar and particularly the postsellar types were more common in older age groups. Median age was highest in the postsellar type and lowest in the conchal type (*p* < 0.001). In unadjusted analyses, all continuous quantitative parameters differed significantly among SS types (all *p* < 0.05). In these unadjusted comparisons, PSA, AD1, AD2, PD1, PD2, SWA, ID, and DST values generally increased from the conchal to the postsellar type. In contrast, TSAW exhibited an inverse pattern, reaching its highest values in the conchal type and its lowest values in the postsellar type (*p* < 0.001). TSFW could only be evaluated in the sellar and postsellar types and was significantly greater in the sellar type than in the postsellar type (*p* < 0.001; Table 3).

Comparison among age groups revealed significant differences in SBA, ASA, PSA, anterior and posterior distances, SWA, DST, ID, ANSTA, and TSAW (all *p* < 0.05). Post hoc analyses showed that anterior and posterior distances, SWA, DST, and ID generally increased with age, although some parameters reached similar values in the older age groups. The main differences in ANSTA were observed between the 0–2-year age group and the older age groups. In contrast, TSAW decreased markedly with increasing age. A weak but statistically significant negative correlation was observed between SBA and age (r = −0.203, *p* = 0.001). Spearman correlation analysis demonstrated significant positive correlations between age and ASA (r = 0.372), PSA (r = 0.299), AD1 (r = 0.850), AD2 (r = 0.904), PD1 (r = 0.815), PD2 (r = 0.899), SWA (r = 0.773), DST (r = 0.661), ID (r = 0.683), and ANSTA(r = 0.322) (all *p* < 0.001). Conversely, TSAW showed a strong negative correlation with age (r = −0.743, *p* < 0.001), whereas TSFW was not significantly correlated with age (r = −0.066, *p* = 0.498; Table 4).

Correlation analysis of SWA demonstrated significant positive correlations with ASA, PSA, AD1, AD2, PD1, PD2, and ANSTA (all *p* < 0.001). TSAW showed a significant negative correlation with SWA (r = −0.633, *p* < 0.001), whereas no significant correlation was found between SWA and TSFW (r = −0.077, *p* = 0.432). Because SWA was calculated as ID × DST, correlations between SWA and ID or DST are mathematically dependent and were not interpreted as independent anatomical associations (Table 5).

## 4. Discussion

Careful preoperative evaluation is essential when planning EETA in pediatric patients, particularly in cases with absent or limited SS pneumatization [16]. Ongoing development of the SS and sinonasal structures throughout childhood leads to considerable anatomical variability, making age-specific radiological assessment important. However, quantitative anatomical data specific to pediatric EETA remain limited in the current literature [17].

Significant positive correlations were identified between SWA, an important parameter related to the available surgical working area during EETA, and ASA, PSA, AD, PD, and ANSTA, whereas TSAW was negatively correlated with SWA (all *p* < 0.001). In our review of the literature, no previous study has directly investigated the relationships among these parameters in a pediatric population. Therefore, our findings suggest that these measurements are closely interrelated from both anatomical and surgical perspectives and contribute novel data to the limited literature in this field.

Haylaz et al. reported a higher mean DST value (mean ± SD: 7.95 ± 1.41) in the 7–18-year age group using lateral radiographs than that observed in our cohort [18]. Magat et al. reported higher DST values in individuals aged 15–19 years than in those aged 9–14 years, consistent with our findings [19]. However, unlike our study, neither investigation identified significant sex-related differences in DST (*p* > 0.05). In a CT-based study of pediatric patients from India, Sawant et al. found that the narrowest ID was observed in the youngest age group (0–6 years), with a mean value of 13.42 ± 2.04 mm [17]. Although their findings were generally consistent with our results, ID values were higher than those observed in our cohort. These differences may be attributable to ethnic and geographic variations between the study populations. Zada et al. evaluated adult patients with and without pituitary lesions and reported no significant difference in ID between individuals without lesions and with small lesions (<10 mm), whereas larger lesions (≥10 mm) were associated with significantly increased ID values [16]. Accordingly, the ID values in our cohort without sellar lesions may be more comparable to those of patients with normal sellar anatomy or small sellar lesions. Previous adult CT-based studies reported higher ID and SWA values in males and higher DST values in females [4,20]. In our cohort, sex-related differences in ID and DST were consistent with these findings; however, SWA did not differ significantly between sexes. The lower ID, DST, and SWA values observed in our pediatric cohort compared with adult populations likely reflect developmental anatomical differences.

In a cross-sectional study of individuals aged 6–42 years, Choi et al. reported age-related increases in the volume and dimensions of the sella turcica [21]. Consistent with these observations, ID, DST, and SWA values increased with age in our cohort. Similarly, Quon et al. demonstrated an age-related increase in ID in pediatric patients who underwent EETA for various sellar and parasellar lesions. Furthermore, a wider ID has been associated with gross total resection in patients with pituitary adenomas [2]. Previous studies have suggested that a narrow ID in younger children may limit surgical maneuverability during EETA [2,17]. In our study, ID, DST, and SWA values were lowest in the 0–2-year age group and highest in the 15–17-year age group (*p* < 0.001). Higher ID and DST values may provide a more favorable anatomical corridor for surgical maneuverability in the horizontal and craniocaudal planes, respectively, whereas larger SWA values may indicate a wider potential working area during EETA. Accordingly, preoperative radiological assessment of ST morphometry may help characterize the anatomical dimensions of the surgical corridor and working space available during the sellar phase of EETA.

Sawant et al. reported that the distance between the external naris and the sella turcica was significantly shorter in children aged 0–6 years than in older age groups (*p* < 0.005) and suggested that this may facilitate surgical access in younger patients [17]. This finding is consistent with our AD1–2 and PD1–2 measurements. In adult populations, Akbudak et al. [20] reported significantly higher AD1–2 and PD1–2 values in males and Arrambide-Garza et al. [4] found significantly higher AD1 and PD1 values in males (both *p* < 0.05). In contrast, no significant sex-related differences were observed in AD1–2 measurements in our pediatric cohort. This discrepancy may be related to sex-dependent differences in sinonasal development during childhood. To our knowledge, this is the first study to evaluate AD2 and PD2 measurements in pediatric patients. In addition, AD1–2 and PD1–2 values were positively correlated with age (all *p* < 0.001). Compared with the 0–2-year age group, AD1–2 and PD1–2 values in the 15–17-year age group were approximately 24–31 mm and 18–24 mm greater, respectively. These findings indicate that the surgical distance to the anterior wall of the sella turcica and the distance to the neurovascular structures located posterior to the sella increase with age. Preoperative radiological assessment of PD1–2 measurements may contribute to the preservation of posterior neurovascular structures during EETA and may help reduce the risk of vascular injury, particularly involving the thalamoperforating arteries. Therefore, consideration of age-related changes in surgical distances may be important when planning EETA in pediatric patients.

Previous pediatric studies have reported a significant age-related increase in SS pneumatization volume, consistent with our findings (*p* < 0.05) [17,22]. Similarly, Ayaz et al. demonstrated an overall increase in SS volume with advancing age in a CT-based pediatric cohort [5]. In a study including both pediatric and adult subjects, Pirinc et al. identified the sellar type as the most common SS type (41.5%), in agreement with our results, and reported lower SS pneumatization volumes in the conchal type and in younger age groups [10]. Consistent with previous adult series, the sellar type was also the most frequent SS type in our pediatric cohort [13,20,23]. In contrast, the prevalence of the conchal type in our exclusively pediatric population (32.7%) was substantially higher than that reported in adult series (2–4%). Doğan et al. reported no significant sex-related differences in SS type distribution (*p* = 0.063) and found that the conchal type was most frequently observed in children and young adults, in line with our findings [24]. Nepal et al. likewise reported a higher prevalence of the conchal type in individuals younger than 10 years of age [25]. In our cohort, 77.1% of conchal SS types were observed in the 0–6-year age groups, whereas 94.4% of postsellar SS types were identified in the 12–17-year age groups. Sellar and postsellar SS types have been reported to provide more favorable anatomical conditions for EETA of the sellar region [1]. Collectively, these findings indicate that SS pneumatization patterns change with age, with the conchal type predominating in early childhood and sellar and postsellar pneumatization becoming increasingly prevalent with advancing age. Furthermore, the increase in SS volume during childhood may facilitate surgical instrument manipulation during EETA. Therefore, preoperative radiological assessment of SS pneumatization patterns may contribute to EETA planning in pediatric patients.

In agreement with our findings, a previous study found significantly greater TSFW values in the sellar type than in the postsellar type, whereas TSAW values were highest in the conchal type among all SS types [20]. The relatively high mean TSAW value and wide interquartile range observed in our study can likely be attributed to the fact that the cohort consisted entirely of pediatric patients; specifically, to the markedly high prevalence of the conchal SS type (86.5%) among children aged 0–6 years and, consequently, to the significantly higher TSAW values observed in this group. Sellar and postsellar SS types are considered more suitable for EETA [26], whereas the conchal SS type is regarded as less favorable for this procedure [27]. This is because, in the sellar and postsellar SS types, the TSAW and TSFW are generally thinner than 1 mm; therefore, access to the sellar dura may be facilitated during surgery in these patients [1]. ST wall thickness varied according to SS type and the degree of pneumatization. However, because SS type showed strong age dependence in this cohort, these unadjusted findings should not be interpreted as an independent effect of SS type on ST wall thickness. In a study with intraoperative correlation, Zada et al. reported that thin sellar walls could often be opened by simple fracture, whereas thicker sellar walls might require more aggressive bone removal techniques, such as drilling or osteotome use. The same authors also emphasized the value of preoperative radiological assessment for anticipating surgical exposure and sellar anatomy [16]. Our age-related findings are consistent with those of Sawant et al. [17], who found greater TSAW values in children aged 0–6 years than in older children (*p* < 0.005). In our cohort, ST wall thickness decreased with increasing SS pneumatization, with the lowest TSAW and TSFW values observed in the postsellar type. In addition, TSAW showed a strong negative correlation with age. These findings suggest that the osseous barrier to the sellar dura may be greatest in the conchal type and least in the postsellar type, with potential implications for preoperative planning in pediatric patients considered for EETA. Therefore, preoperative CECT-based assessment of ST wall thickness may help anticipate surgical difficulty and guide the selection of an appropriate bone removal technique.

In a pediatric study, Higashino et al. reported higher SBA values in females than in males, although SBA remained relatively stable across age groups [22]. Similar to previous studies in adults, type B was the most common SBA pattern in our pediatric cohort (64.2%), with prevalences ranging from 69% to 80% in adult series [4,15,20]. Furthermore, SBA values were significantly higher in females than in males and were highest in the 0–2-year age group in our cohort. Compared with the other SBA types, type B morphology has been suggested to provide more favorable anatomical conditions for adequate exposure and resection of pituitary lesions during EETA [4,15].

Zhang et al. found no significant sex-related differences in ANSTA values in a study including both pediatric and adult patients [14]. Arrambide-Garza et al. [4] found no significant sex-related differences in ASA or PSA values and Akbudak et al. [20] documented no significant difference in PSA values but significantly greater ASA and ANSTA values in females than in males (*p* < 0.05). In our cohort, no significant sex-related differences were identified for ASA or PSA, whereas ANSTA values were significantly greater in females than in males. Consistent with the findings of Zhang et al. [14], ANSTA values were lower in the younger age groups in our study. In addition, ASA and PSA generally increased with age. Based on our review of the literature, age-related variations in ASA and PSA have not previously been reported in pediatric populations. Higher ASA and PSA values may facilitate instrument manipulation, particularly in the craniocaudal direction, during EETA and thereby enhance surgical maneuverability.

Among the evaluated parameters, SS type, ID, DST, SWA, and ST wall thickness may be prioritized for routine preoperative assessment because they are clinically relevant and relatively easy to measure on CECT. SS type provides a rapid overview of sphenoid sinus pneumatization. ID reflects the transverse width of the surgical corridor between the internal carotid arteries; DST reflects the craniocaudal depth of the sellar surgical corridor; therefore SWA may help estimate the available working area during the sellar phase of EETA. In addition, TSAW and TSFW may help anticipate the thickness of the osseous barrier overlying the sellar dura. Overall, these parameters may provide practical anatomical information for anticipating technical difficulty, optimizing surgical trajectory, and supporting individualized EETA planning in pediatric patients.

Several limitations of this study should be acknowledged. First, this was a single-center retrospective study, which may limit the generalizability of the findings. Second, potential confounding factors should be considered. Although age and sex were evaluated in the present study, anthropometric variables such as height, weight, body mass index, and head circumference, as well as detailed clinical symptoms, were not available for all patients because of the retrospective study design and therefore could not be included in additional analyses. Because SS type was strongly age-dependent, differences in measured parameters among SS types should be interpreted as unadjusted associations rather than independent effects. Third, because EETA was not performed in the study cohort, surgical or intraoperative validation of the measured parameters could not be performed. Future multicenter studies including operative findings, surgical outcomes, and external validation cohorts are needed to confirm the clinical utility and generalizability of these CECT-based measurements. Fourth, because the measured parameters were obtained from normal ST anatomy, the reported parameters may be particularly applicable to EETA planning in lesions that do not substantially alter the anatomical configuration, such as microadenomas. Nevertheless, although large sellar lesions may modify these measurements through anatomical expansion, the altered parameters can still be readily assessed and remeasured on CECT images.

## 5. Conclusions

Our study demonstrates substantial age-related changes in SS and ST anatomy in pediatric patients. SS pneumatization increased with age, accompanied by a progressive rise in the prevalence of the sellar and postsellar SS types. Surgical angles, distances, ANSTA, SWA, DST, and ID increased with advancing age, whereas TSAW decreased markedly. These findings suggest that, with the exception of AD and PD measurements, anatomical parameters relevant to the surgical corridor and safe working area for EETA generally reach more favorable values in older pediatric age groups.

In particular, the narrower ID, shorter surgical distances, smaller SWA and lower DST observed in children aged 0–6 years may complicate the use of standard surgical instruments designed for adults. Therefore, preoperative CECT-based assessment of sinonasal and sellar anatomy and sella turcica-related quantitative parameters may provide useful information for surgical planning and appropriate instrument selection in pediatric patients considered for EETA. In turn, this imaging-based approach may also inform strategies aimed at reducing complication risk during EETA.

## Figures and Tables

**Table 1:** Descriptive Statistics of Age and Measured Parameters for All Patients.

Parameter	n	Mean ± SD	Median (IQR)
Age (years)	254	9.72 ± 5.03	10 (6–14)
Skull base angle (°)	254	114.70 ± 8.02	114.6 (109.1–119.2)
Anterior surgical angle (°)	254	30.74 ± 3.19	30.6 (28.7–32.9)
Posterior surgical angle (°)	254	22.13 ± 3.09	22.1 (20.1–24.2)
Anterior distance 1 (mm)	254	66.04 ± 11.26	67.7 (59.3–74.5)
Anterior distance 2 (mm)	254	82.49 ± 14.06	85.3 (73.6–93.1)
Posterior distance 1 (mm)	254	78.23 ± 9.58	79.0 (71.9–85.0)
Posterior distance 2 (mm)	254	94.82 ± 11.95	95.2 (87.2–103.5)
Safe window area (mm^2^)	254	86.68 ± 28.01	86.7 (68.3–106.3)
Depth of the sella turcica (mm)	254	6.36 ± 1.25	6.5 (5.6–7.1)
Intercarotid distance (mm)	254	13.38 ± 2.68	13.2 (11.4–15.5)
Apex nasi–sella turcica angle (°)	254	6.38 ± 0.95	6.3 (5.7–7.0)
Thickness of the sella turcica anterior wall (mm)	254	3.16 ± 3.36	1.1 (0.9–5.8)
Thickness of the sella turcica floor wall (mm)	107	0.90 ± 0.16	0.9 (0.8–1.0)

SD, standard deviation; IQR, interquartile range.

**Table 2:** Comparison of Age Distribution, Sphenoid Sinus Types, and Measured Parameters According to Sex.

Variable	Male (n = 145)	Female (n = 109)	*p* Value
Age group, n (%)			0.761 *
0–2 years	21 (14.5)	12 (11.0)	
3–6 years	24 (16.6)	17 (15.6)	
7–11 years	45 (31.0)	34 (31.2)	
12–14 years	21 (14.5)	22 (20.2)	
15–17 years	34 (23.4)	24 (22.0)	
Sphenoid sinus type, n (%)			0.138 *
Conchal type	53 (36.6)	30 (27.5)	
Presellar type	35 (24.1)	29 (26.6)	
Sellar type	44 (30.3)	45 (41.3)	
Postsellar type	13 (9.0)	5 (4.6)	
Continuous variables, Median (IQR)			*p* **
Age (years)	10 (5–14)	11 (6–14)	0.354
Skull base angle (°)	113.0 (107.5–117.9)	116.5 (111.9–122.1)	<0.001
Anterior surgical angle (°)	30.2 (28.5–32.4)	31.3 (29.0–33.2)	0.079
Posterior surgical angle (°)	22.1 (20.1–24.2)	22.1 (19.9–24.2)	0.928
Anterior distance 1 (mm)	69.1 (58.0–76.6)	67.0 (61.3–73.1)	0.147
Anterior distance 2 (mm)	85.5 (72.7–97.1)	85.0 (75.3–90.2)	0.146
Posterior distance 1 (mm)	81.2 (71.8–87.8)	77.5 (72.0–83.1)	0.005
Posterior distance 2 (mm)	96.3 (87.6–107.3)	94.8 (87.0–100.6)	0.033
Safe window area (mm^2^)	89.0 (68.3–111.2)	84.0 (68.6–102.9)	0.314
Depth of the sella turcica (mm)	6.4 (5.6–7.0)	6.7 (5.9–7.4)	0.037
Intercarotid distance (mm)	13.6 (12.2–16.2)	12.5 (11.1–14.6)	0.001
Apex nasi–sella turcica angle (°)	6.0 (5.6–6.6)	6.7 (6.2–7.4)	<0.001
Thickness of the sella turcica anterior wall (mm)	1.2 (0.9–6.9)	1.0 (0.85–3.7)	0.155
Thickness of the sella turcica floor wall (mm) †	0.9 (0.8–1.0)	0.9 (0.8–1.0)	0.789

* Pearson chi-square test. ** Mann–Whitney U test. † Thickness of the sella turcica floor wall was measured only in patients with sellar and postsellar sphenoid sinus types. A *p* value < 0.05 was considered statistically significant. IQR, interquartile range.

**Table 3:** Comparison of Age Distribution and Measured Parameters According to Sphenoid Sinus Types.

Variable	Conchal Type (n = 83)	Presellar Type (n = 64)	Sellar Type (n = 89)	Postsellar Type (n = 18)	*p* Value
Age group, n (%)					<0.001 *
0–2 years	32 (38.6)	1 (1.6)	0 (0.0)	0 (0.0)	
3–6 years	32 (38.6)	8 (12.5)	1 (1.1)	0 (0.0)	
7–11 years	17 (20.5)	39 (60.9)	22 (24.7)	1 (5.6)	
12–14 years	1 (1.2)	6 (9.4)	28 (31.5)	8 (44.4)	
15–17 years	1 (1.2)	10 (15.6)	38 (42.7)	9 (50.0)	
Continuous variables, Median (IQR)					*p* **
Age (years)	4 (2–6) ^a^	9.5 (7.3–11.8) ^b^	14 (11–16) ^c^	15 (12.8–17) ^d^	<0.001
Skull base angle (°)	115.4 (110.3–122.0)	115.1 (108.9–119.8)	113.5 (107.6–118.4)	111.9 (106.7–115.1)	0.038
Anterior surgical angle (°)	28.5 (26.3–30.9) ^a^	31.5 (29.9–33.2) ^b^	31.5 (29.9–33.4) ^b^	30.4 (29.6–33.6) ^b^	<0.001
Posterior surgical angle (°)	20.2 (18.2–22.4) ^a^	22.5 (21.4–24.2) ^b^	22.5 (21.2–24.6) ^b^	23.1 (20.2–25.0) ^b^	<0.001
Anterior distance 1 (mm)	53.0 (48.0–60.2) ^a^	68.8 (64.5–72.4) ^b^	73.8 (70.0–77.0) ^c^	77.2 (74.4–80.5) ^d^	<0.001
Anterior distance 2 (mm)	66.5 (61.2–74.8) ^a^	84.9 (79.0–89.0) ^b^	92.0 (88.0–97.3) ^c^	97.7 (92.0–102.4) ^d^	<0.001
Posterior distance 1 (mm)	69.2 (65.2–75.3) ^a^	78.5 (74.6–83.1) ^b^	83.7 (79.6–87.5) ^c^	87.8 (84.9–91.1) ^d^	<0.001
Posterior distance 2 (mm)	83.4 (78.5–90.7) ^a^	93.9 (89.0–100.2) ^b^	102.9 (97.6–107.8) ^c^	107.7 (102.0–112.9) ^d^	<0.001
Safe window area (mm^2^)	60.3 (44.6–74.3) ^a^	88.9 (78.8–103.0) ^b^	101.1 (87.7–114.1) ^c^	112.4 (98.3–124.3) ^d^	<0.001
Depth of the sella turcica (mm)	5.3 (4.5–5.9) ^a^	6.8 (6.2–7.2) ^b^	6.8 (6.3–7.5) ^b^	7.0 (6.6–7.3) ^b^	<0.001
Intercarotid distance (mm)	11.1 (9.8–12.4) ^a^	13.2 (11.9–15.0) ^b^	14.8 (13.2–16.4) ^c^	16.1 (14.3–17.1) ^d^	<0.001
Apex nasi–sella turcica angle (°)	5.9 (5.2–6.4) ^a^	6.7 (6.1–7.2) ^b^	6.5 (6.0–7.2) ^b^	6.1 (5.9–6.6) ^ab^	<0.001
Thickness of the sella turcica anterior wall (mm)	8.1 (6.0–9.2) ^d^	1.2 (1.0–1.4) ^c^	0.9 (0.8–1.0) ^b^	0.8 (0.7–0.8) ^a^	<0.001
Thickness of the sella turcica floor wall (mm) †	–	–	0.9 (0.8–1.0)	0.7 (0.7–0.8)	<0.001

* Pearson chi-square test; ** Kruskal–Wallis test; † Comparison between sellar and postsellar sphenoid sinus types was performed using the Mann–Whitney U test. A *p* value < 0.05 was considered statistically significant. ^abcd^ Groups with different superscript letters differed significantly in post hoc pairwise comparisons. IQR, interquartile range.

**Table 4:** Comparison of Measured Parameters According to Age Group.

Variable	0–2 Years	3–6 Years	7–11 Years	12–14 Years	15–17 Years	*p* *	r	*p* **
Skull base angle (°)	118.6 (112.5–125.3) ^a^	115.0 (109.3–120.7) ^ab^	114.3 (107.5–119.5) ^b^	115.3 (109.0–118.0) ^ab^	112.7 (107.4–117.1) ^b^	0.007	−0.203	0.001
Anterior surgical angle (°)	26.3 (25.0–29.2) ^a^	29.7 (27.6–32.0) ^b^	31.2 (29.6–33.2) ^bc^	31.6 (29.7–33.9) ^c^	31.3 (29.6–33.1) ^c^	<0.001	0.372	<0.001
Posterior surgical angle (°)	19.0 (16.9–21.2) ^a^	21.7 (19.6–23.3) ^b^	22.5 (21.5–24.2) ^bc^	23.0 (21.3–25.1) ^c^	22.2 (20.8–24.7) ^bc^	<0.001	0.299	<0.001
Anterior distance 1 (mm)	46.8 (40.8–49.7) ^a^	57.6 (53.6–61.8) ^b^	67.1 (64.0–71.5) ^c^	74.1 (70.3–77.3) ^d^	75.8 (72.3–80.7) ^d^	<0.001	0.850	<0.001
Anterior distance 2 (mm)	56.7 (52.4–62.9) ^a^	71.6 (68.2–75.1) ^b^	83.0 (79.0–88.0) ^c^	92.0 (87.6–95.9) ^d^	96.7 (91.3–101.7) ^e^	<0.001	0.904	<0.001
Posterior distance 1 (mm)	64.0 (57.9–67.3) ^a^	71.8 (68.0–74.2) ^b^	78.5 (75.0–82.1) ^c^	84.2 (81.0–87.5) ^d^	87.5 (82.8–92.1) ^d^	<0.001	0.815	<0.001
Posterior distance 2 (mm)	78.2 (69.3–81.6) ^a^	86.4 (82.9–88.7) ^b^	94.2 (90.5–98.7) ^c^	102.6 (98.6–105.9) ^d^	107.3 (102.5–112.3) ^e^	<0.001	0.899	<0.001
Safe window area (mm^2^)	44.1 (36.6–50.2) ^a^	68.2 (57.0–76.3) ^b^	83.9 (74.6–94.2) ^c^	100.8 (91.5–112.6) ^d^	113.9 (98.4–126.1) ^e^	<0.001	0.773	<0.001
Depth of the sella turcica (mm)	4.5 (3.8–4.9) ^a^	5.9 (5.2–6.5) ^b^	6.5 (5.9–6.9) ^c^	6.9 (6.4–7.5) ^d^	7.2 (6.5–7.7) ^d^	<0.001	0.661	<0.001
Intercarotid distance (mm)	9.8 (8.8–10.8) ^a^	11.7 (10.7–12.7) ^b^	13.2 (12.1–14.6) ^c^	14.6 (13.2–16.2) ^d^	15.9 (13.9–17.0) ^d^	<0.001	0.683	<0.001
Apex nasi–sella turcica angle (°)	5.4 (4.8–6.1) ^a^	6.4 (5.7–7.0) ^b^	6.4 (5.9–6.9) ^b^	6.5 (6.0–7.2) ^b^	6.6 (6.0–7.2) ^b^	<0.001	0.322	<0.001
Thickness of the sella turcica anterior wall (mm)	9.2 (8.3–10.1) ^a^	6.0 (3.0–8.2) ^b^	1.2 (0.9–1.9) ^c^	0.9 (0.8–1.0) ^d^	0.9 (0.8–1.0) ^d^	<0.001	−0.743	<0.001
Thickness of the sella turcica floor wall (mm) ‡	—	—	0.9 (0.8–1.0)	0.9 (0.73–1.0)	0.9 (0.8–1.0)	0.700	−0.066	0.498

* Kruskal–Wallis test. ** Spearman correlation analysis. A *p* value < 0.05 was considered statistically significant. ^abcde^ Different superscript letters indicate statistically significant differences in post hoc pairwise comparisons (Bonferroni-adjusted *p* ≤ 0.005). ‡ Thickness of the sella turcica floor wall could not be measured in the 0–2-year age group and was excluded from analysis because it was identified in only one patient in the 3–6-year age group.

**Table 5:** Spearman Correlations Between Safe Window Area and Other Measured Parameters.

Parameter	r	*p* Value	Parameter	r	*p* Value
Skull base angle	−0.163	0.009	Posterior distance 1	0.705	<0.001
Anterior surgical angle	0.352	<0.001	Posterior distance 2	0.760	<0.001
Posterior surgical angle	0.273	<0.001	Depth of the sella turcica †	0.811	<0.001
Anterior distance 1	0.725	<0.001	Intercarotid distance †	0.863	<0.001
Anterior distance 2	0.766	<0.001	Apex nasi–sella turcica angle	0.341	<0.001
Thickness of the sella turcica anterior wall	−0.633	<0.001	Thickness of the sella turcica floor wall	−0.077	0.432

r, Spearman correlation coefficient. Correlations with r ≥ 0.70 were considered strong. A *p* value < 0.05 was considered statistically significant. † Because SWA was calculated as ID × DST, correlations between SWA and ID or DST are mathematically dependent and should not be interpreted as independent anatomical associations.

## Data Availability

The data that support the findings of this study are available from the corresponding author upon reasonable request. Due to privacy and ethical restrictions, the data are not publicly available.
